# IGFBP-3 Inhibits Cytokine-Induced Insulin Resistance and Early Manifestations of Atherosclerosis

**DOI:** 10.1371/journal.pone.0055084

**Published:** 2013-01-28

**Authors:** Lathika Mohanraj, Ho-Seong Kim, Wei Li, Qing Cai, Ki Eun Kim, Hye-Jung Shin, Yong-Jae Lee, Woo Jung Lee, Jung Hyun Kim, Youngman Oh

**Affiliations:** 1 Department of Pathology, Virginia Commonwealth University, Richmond, Virginia, United States of America; 2 Department of Pediatrics, Institute of Endocrinology, Yonsei University College of Medicine, Seoul, Korea; 3 Biocure Pharma LLC, Richmond, Virginia, United States of America; 4 Department of Pediatrics, CHA University College of Medicine, Seoul, Korea; 5 Department of Pediatrics, National Medical Center, Seoul, Korea; 6 Department of Family Medicine, Yonsei University College of Medicine, Seoul, Korea; Virginia Tech, United States of America

## Abstract

Metabolic syndrome is associated with visceral obesity, insulin resistance and an increased risk of cardiovascular diseases. Visceral fat tissue primarily consists of adipocytes that secrete cytokines leading to a state of systemic inflammation in obese conditions. One of the IGF-independent functions of IGFBP-3 is its role as an anti-inflammatory molecule. Our study in obese adolescents show a decrease in total IGFBP-3 levels and increase in proteolyzed IGFBP-3 in circulation when compared to their normal counterparts and establishes a positive correlation between IGFBP-3 proteolysis and adiposity parameters as well as insulin resistance. In human adipocytes, we show that IGFBP-3 inhibits TNF-α-induced NF-κB activity in an IGF-independent manner, thereby restoring the deregulated insulin signaling and negating TNF-α-induced inhibition of glucose uptake. IGFBP-3 further inhibits TNF-α, CRP and high glucose-induced NF-κB activity in human aortic endothelial cells (HAECs) and subsequently suppresses monocyte adhesion to HAEC through the IGFBP-3 receptor. In conclusion, these findings suggest that reduced levels of IGFBP-3 in circulation and reduced expression of IGFBP-3 in macrophages in obesity may result in suppression of its anti-inflammatory functions and therefore IGFBP-3 may present itself as a therapeutic for obesity-induced insulin resistance and for events occurring in the early stages of atherosclerosis.

## Introduction

Over the past two decades obesity has dramatically increased resulting in one-third of the adults in the United States being obese [1]. Obesity is a complex disorder and is a major risk factor associated with the incidence of diabetes, insulin resistance, cardiovascular diseases (CVD), hypertension, diabetic retinopathy and other metabolic disorders [2]. The endocrine paradigm suggests that visceral fat in obesity, consisting primarily of adipocytes, secretes various pro-inflammatory and pro-atherogenic adipokines such as TNF-α, C-reactive protein (CRP) [3], IL-6 and others [4] creating a state of local inflammation further resulting in chronic systemic inflammation and accelerating the events leading to metabolic disorders.

The IGF system plays a major role in growth, development and maintenance of homeostasis in normal cells. Insulin-like growth factor binding protein-3 (IGFBP-3), the major binding protein in circulation [5] has been shown to be associated with CVD, coronary events [6] and thickness of the intima-media [7]. A study comparing the concentrations of IGFs and IGFBP-3 with respect to BMI and body fat revealed that these levels are decreased in obese women [8]. IGFBP-3 overexpression in the retinal endothelium has also been shown to restore vascular integrity, suggesting that IGFBP-3 may represent treatment of diabetic retinopathy [9]. These observations clearly indicate involvement of IGFBP-3 in CVD, obesity and insulin resistance; however the mechanisms responsible for the role of IGFBP-3 in metabolic syndrome remains poorly understood.

In addition to IGF-dependent functions, IGF-independent functions of IGFBP-3 have also been studied [10]. Some of the disease states that show IGF-independent actions of IGFBP-3 include cancer [11] and asthma [12]. IGFBP-3 exerts antitumor and anti-inflammatory effects via a specific receptor (IGFBP-3R) that involves activation of caspase pathway and cross-talk with NF-κB signaling [11], [13]. Another interesting attribute that may regulate the functions of IGFBP-3 is proteolytic cleavage of IGFBP-3 that has been reported in several physiological and pathological conditions, including cancer, type 1 and 2 diabetes, burn injuries, and surgery, suggesting that catabolic states increase degradation of circulating IGFBP-3 [14]–[18]. In line with the potential anti-inflammatory role of IGFBP-3 in cancers and other diseases [11]–[13], [15], [16] and its crosstalk with the NF-κB pathway, our study investigates the relationship between proteolyzed IGFBP-3 in circulation and the parameters of adiposity and the potential role of IGFBP-3 in obesity-induced insulin resistance and its involvement in the progression of atherosclerosis and CVD.

## Results

### IGFBP-3 Profile in Normal and Obese Adolescents

**Figure 1:**
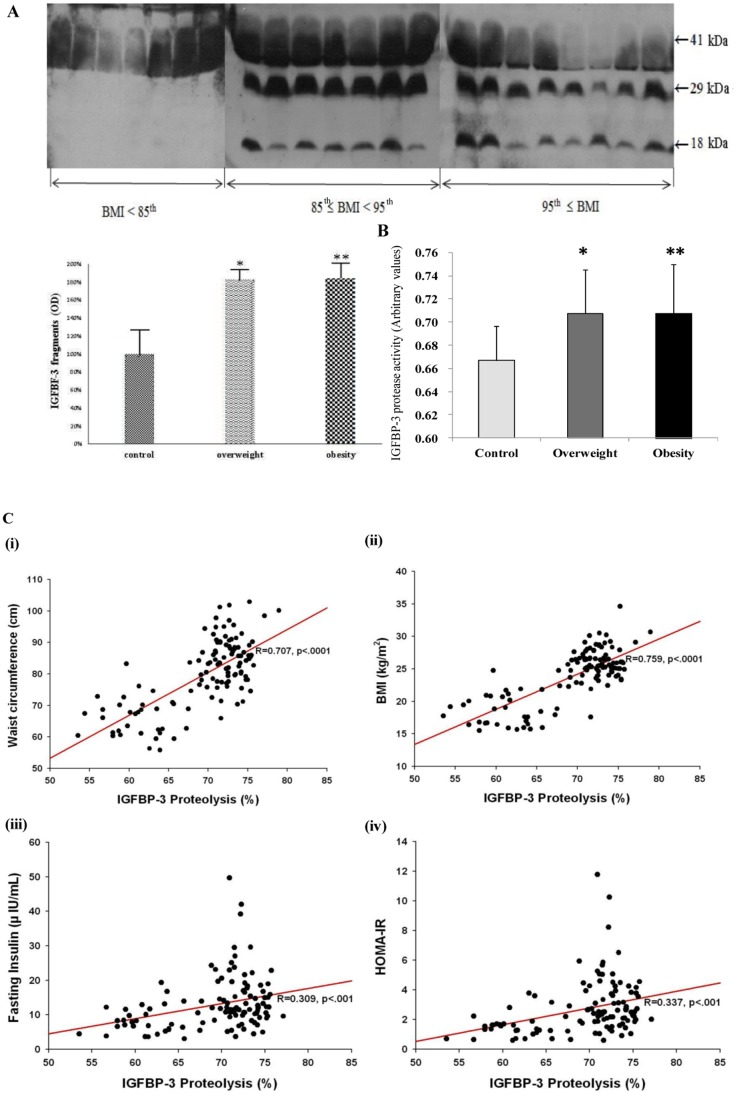
Increased IGFBP-3 proteolysis in obese adolescents. (A) The IGFBP-3 proteolytic fragments were detected by Western blot assay. Optical density was significantly increased in the overweight and obesity. *, control vs. overweight, 2.09±1.32 (100%) vs. 3.83±0.37 (183%), P<0.001; **, control vs. obesity, 2.09±1.32 (100%) vs. 3.85±0.63 (184%), P<0.001). (B) Protease activity assay was performed to determine IGFBP-3 proteolysis. The activity was measured as optical density and it was significantly higher in the overweight and obese group. *, control vs. overweight, 0.667±0.030 vs. 0.707±0.038, P = 0.001; **, control vs. obesity, 0.667±0.030 vs. 0.708±0.043, P = 0.002). (C) Relationship between IGFBP-3 proteolysis and (i) waist circumference (r = 0.707, P<0.0001), (ii) BMI (r = 0.759, P<0.0001), (iii) fasting insulin (r = 0.309, P<0.001), and (iv) HOMA-IR (r = 0.337, P<0.001) were calculated by Pearson’s correlation coefficient.

A total of 197 adolescents aged 12 to 13 years were classified into control, overweight and obese groups according to BMI for age and gender. Comparisons of clinical features in all groups (Table 1) show that the obese group had significantly higher height, weight, BMI, waist circumference, systolic BP and diastolic BP than controls. HDL-cholesterol levels were markedly decreased in the obesity group. The obesity group had much higher levels of triglycerides, LDL-cholesterol and ALT than the controls (Table 2). Fasting insulin levels differed significantly in the obesity compared with other groups. The levels of fasting glucose were increased in the overweight and obesity compared to the control but was not significantly different between each other. HOMA-IR increased similarly (Table 3). Serum IGF-1 was increased in overweight and obese individuals compared with the normal group whereas total IGFBP-3 showed a slight increase in overweight individuals. However both IGF-1 and total IGFBP-3 levels showed a significant decrease in obese individuals compared to overweight individuals. Total IGFBP-3 levels in particular were less even when compared to the normal group (Table 1). The data further shows an increase in the IGF-1/IGFBP-3 in both overweight and obese groups when compared to the control group. In order to further analyze the IGFBP-3 levels, we determined the presence and distribution of proteolyzed IGFBP-3 in the overweight and obese subjects by Western immunoblot assay as well as protease activity assay. The western blot analysis of the serum samples shows a robust presence of IGFBP-3 proteolytic fragments (29-kDa and 18-kDa) in the obese and overweight groups, but low or no detection of these fragments were seen in controls. Densitometric analysis of the proteolytic IGFBP-3 fragments showed a 183 percent and a 184 percent increase in the overweight and obese groups respectively when compared to the control group (Figure 1A).

**Table 1:** Clinical and anthropometric characteristics of subjects.

	Controls	Overweight	Obese	*P* value
n (M:F)	100 (49∶51)	41 (19∶22)	56 (25∶31)	NS
Age (years)	12.2±0.4	12.2±0.4	12.2±0.3	NS
Weight (kg)	47.2±6.9	61.0±6.9	69.7±7.7	a
Height (cm)	156.9±7.1	157.9±7.2	160±5.7	b
BMI (kg/m^ 2^)	19.1±1.9	24.4±1.0	27.2±1.8	a
Waist Circumference (cm)	68.5±6.2	80.8±5.6	87.9±7.0	a
Systolic BP (mmHg)	101.2±10.8	107.9±10.6	114.8±10.7	a
Diastolic BP (mmHg)	63.6±6.4	65.6±7.5	69.9±7.8	c
IGF-1 (ng/ml)	120.3±63.4	153.6±88.1	145.5±73.8	d
IGFBP-3 (ug/mL)	7.11±1.16	7.47±1.02	6.82±1.28	e
IGF-1/IGFBP-3 molar ratio	0.129±0.051	0.162±0.068	0.161±0.08	f

NS, not significant; BMI, body mass index. Values are expressed as mean ± SD.

Each group was classified by the BMI percentile according to 2007 Korea Growth Charts.

a control *vs*. overweight; overweight *vs*. obesity; control *vs*. obesity; respectively, *P*<0.01.

b control *vs*. overweight (*P*<0.01), and overweight *vs*. obesity (*P*<0.01).

c control *vs*. overweight (*P*<0.01), and control *vs*. obesity (*P*<0.01).

d control *vs*. overweight (*P*<0.05), and control *vs*. obesity (*P*<0.05).

e overweight *vs*. obese (*P*<0.05).

f control *vs*. overweight (*P* **<**0.004), and control *vs*. obesity (*P*<0.01).

**Table 2:** Biochemical characteristics of subjects.

	Controls	Overweight	Obese	*P* value
Total cholesterol (mg/dL)	145.4±32.3	158.6±29.0	150.3±34.0	NS
HDL-cholesterol (mg/dL)	53.1±13.3	53.6±11.4	46.5±10.6	a
Triglycerides (mg/dL)	82.9±37.6	110.9±47.7	103.2±36.8	b
LDL-cholesterol (mg/dL)	75.9±24.2	82.9±23.3	84.7±26.5	c
AST (IU/L)	21.8±12.6	22.9±10.3	26.0±17.6	NS
ALT (IU/L)	11.4±21.4	17.2±2.7	20.9±20.8	a
hs-CRP (mg/dL)	0.07±0.13	0.07±0.13	0.11±0.11	NS

Values are expressed as mean ± SD.

a control *vs*. obesity (*P*<0.01), and overweight *vs*. obesity (*P*<0.01).

b control *vs*. overweight (*P*<0.01), and control *vs*. obesity (*P*<0.01).

c control *vs*. overweight (*P*<0.05), and control *vs*. obesity (*P*<0.05).

*P* values were calculated by one-way ANOVA.

**Table 3:** Insulin resistance of subjects.

	Controls	Overweight	Obese	*P* value
Fasting glucose (mg/dL)	75.8±11.3	86.3±12.5	85.3±7.2	a
Fasting insulin (µIU/Ml)	8.0±4.0	12.9±8.7	16.9±9.5	b
HOMA-IR	1.5±0.8	2.9±3.2	3.6±2.2	a

a control *vs*. overweight (*P*<0.001), and control *vs*. obesity (*P*<0.001).

b control *vs*. overweight, overweight *vs*. obesity, and control *vs*. obesity, respectively; *P*<0.001.

*P* values were calculated by one-way ANOVA.

To investigate this further protease activity assay was performed on the samples, in which recombinant human glycosylated IGFBP-3 was biotinylated and IGFBP-3 protease activity was measured as the ability of serum samples from control, overweight and obese individuals to proteolyze biotinylated IGFBP-3. Results show that IGFBP-3 protease activity increased in overweight and obese individuals compared to the control group, but did not differ between the overweight and obese groups (Figure 1B).

Further statistical analyses revealed that proteolyzed IGFBP-3 positively correlates with adiposity parameters such as waist circumference (r = 0.608, P<0.001), BMI (r = 0.4651, P<0.001), fasting insulin (r = 0.307, P = 0.001), and insulin resistance index (HOMA-IR) (r = 0.313, P<0.001) in overweight and obese population (Figure 1C), suggesting that predisposition to insulin resistance and other consequences of increased visceral fat can occur even in overweight individuals and not necessarily only in obese population.

Taken together, based on our data and previous studies demonstrating IGFBP-3 proteolysis in patients with type 2 diabetes [17], we speculate that increased proteolytic IGFBP-3 fragments in overweight and obese individuals probably result in reduced levels of intact IGFBP-3 thereby inhibiting its IGF-independent anti-inflammatory functions. Based on these findings, we further investigated the effect of IGFBP-3 and its underlying mechanism in cytokine-induced insulin resistance in adipocytes and early manifestations of atherosclerosis in HAECs.

### TNF-α Activates the NF-κB Pathway in Adipocytes in an *in vitro* System

**Figure 2:**
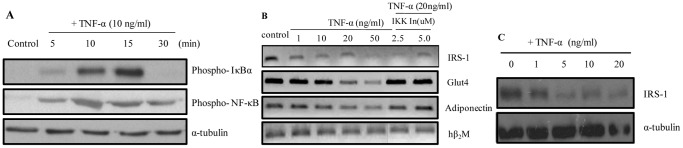
Effect of TNF-α on NF-κB signaling in adipocytes. (A) Differentiated adipocytes were treated with 10 ng/ml TNF-α for 0, 5, 10, 15 and 30 minutes respectively and its effect on the levels of phospho-IκBα and phospho-NF-κB was detected by western blotting. α-tubulin served as the loading control. (B) Differentiated adipocytes were treated with different concentration (1, 10, 20 and 50 ng/ml) of TNF-α for 18 hours followed by analysis of the mRNA levels of IRS-1, GLUT4 and adiponectin by RT-PCR. Cells were treated with 2.5 and 5.0 µM of IKK-IV inhibitor followed by treatment with 20 ng/ml TNF-α. hβ2M was used as the internal control for normalizing mRNA levels. (C) Cells were treated with 1, 5, 10 and 20 ng/ml TNF-α for 24 hour and immunoblotted for IRS-1 and α-tubulin.

Visceral adipocytes are highly responsive to TNF-α mediated activation of NF-κB pathway [18]. In order to confirm that the NF-κB pathway was intact in fully differentiated human primary adipocytes (ASCs), cells were treated with TNF-α and the pattern of NF-κB activation was analyzed. Figure 2A clearly indicates the cyclic phosphorylation pattern of IκBα and a time dependent increase in the levels of phospho-NF-κBp65. These results indicate that TNF-α activates the NF-κB pathway in ASCs.

### Activation of the NF-κB Pathway Affects Insulin Signaling in Adipocytes

Cytokines such as TNF-α, interferes with insulin signaling by decreasing the levels of Insulin receptor substrate-1 (IRS-1) and Glucose transporter-4 (GLUT4) [19], [20]. Deficient levels of adiponectin, an insulin sensitizing and anti-inflammatory adipokine are observed in individuals with visceral fat [21]. Our experiment was to determine the effect of TNF-α on IRS-1, GLUT4 and adiponectin levels in *in vitro* ASCs. Figure 2B demonstrates that TNF-α treatment decreases the transcriptional levels of IRS-1, GLUT4 and adiponectin in a dose dependent manner and treatment with IKK inhibitor shows that the reduced mRNA levels of our genes of interest were restored, confirming that TNF-α exerts its effect through the NF-κB pathway in adipocytes. This was confirmed by the decrease in the protein levels of IRS-1 with dose dependent TNF-α treatment (Figure 2C).

### IGFBP-3 Inhibits the NF-κB Activity in Primary Human Adipocytes

**Figure 3:**
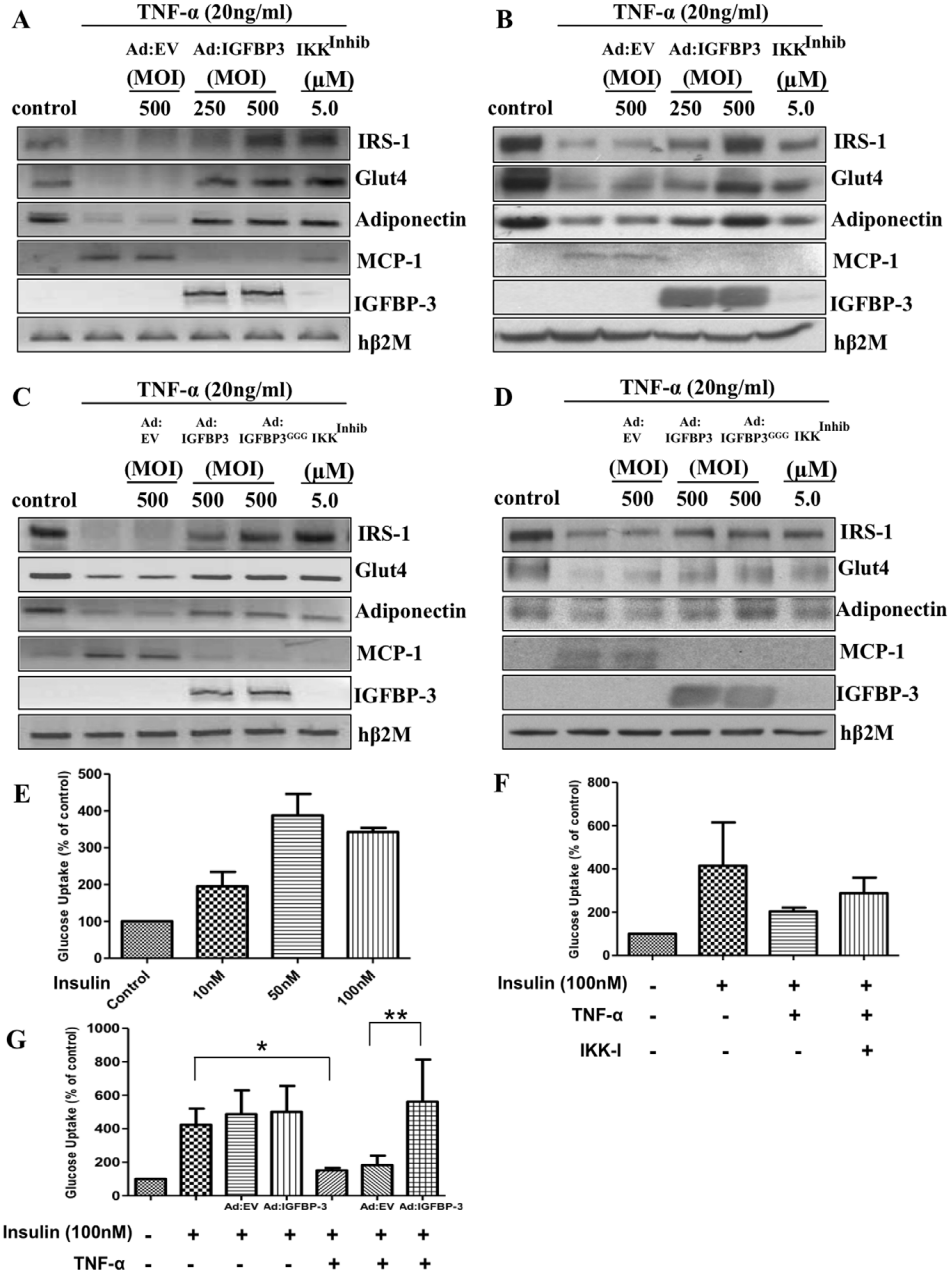
IGFBP-3 inhibits the NF-κB pathway in primary human adipocytes in an IGF-independent manner. Effect of IGFBP-3 on IRS-1, GLUT4, adiponectin and MCP-1 levels induced upon treatments with TNF-α (20 ng/ml) was analyzed by (A) RT-PCR (18 hours) and (B) immunoblot analysis (36 hours). Similar experiments were performed by infecting adipocytes with mutant-Ad:IGFBP3GGG followed by treatment with TNF-α and analyzing IRS-1, GLUT4, adiponectin and MCP-1 levels by (C) RT-PCR and (D) immunoblotting. Overexpressed IGFBP-3 levels were confirmed in these experiments. Cells were infected with either Ad:EV (m.o.i. 500), Ad:IGFBP-3 (m.o.i. 250, 500), Ad:IGFBP-3GGG (m.o.i. 500) or IKK IV inhibitor at a concentration of 5.0 µM. (E) Differentiated adipocytes were treated with 10, 50 and 100 nM of insulin and a glucose uptake assay was performed on these cells. (F) The inhibitory effect of TNF-α (20 ng/ml) alone and in the presence of IKK IV inhibitor on the biological functions of insulin was analyzed by the glucose uptake assay. (G) Human adipocytes were treated with TNF-α in the presence of insulin (100 nM) and the insulin sensitizing effect of IGFBP-3 was analyzed by glucose uptake assay. Cells were infected with either Ad:EV or Ad:IGFBP-3 (m.o.i. 250), n = 3, in duplicate; *, p<0.001;**, p<0.05.

To investigate the anti-inflammatory function of IGFBP-3 on TNF-α-induced NF-κB pathway and insulin signaling in ASCs, TNF-α treated cells were infected with adenoviral constructs containing IGFBP-3 sequence (Ad:IGFBP-3). ASCs produce no detectable basal level of IGFBP-3 and TNF-α-treatment shows no change in IGFBP-3 at the mRNA and protein levels (Figure 3). TNF-α inhibits IRS-1, GLUT4 and adiponectin in TNF-α-treated control cells, however, infection with Ad:IGFBP-3 restored IRS-1, GLUT4 and adiponectin mRNA (Figure 3A) and protein levels (Figure 3B). Overexpressed IGFBP-3 inhibits TNF-α-mediated induction of MCP-1 at both mRNA (Figure 3A) and protein levels (Figure 3B). Cells treated with IKK inhibitor confirmed involvement of the NF-κB pathway. These data show that IGFBP-3 inhibits the TNF-α-induced NF-κB activity, thereby restoring insulin signaling in ASCs.

### IGFBP-3 Inhibits NF-κB Pathway in Human Adipocytes in an IGF-independent Manner

In order to decipher if IGFBP-3 inhibits the NF-κB pathway in adipocytes in an IGF- independent manner, we used IGFBP-3 ^GGG^ mutant, which has no binding affinity to IGF and therefore any effect of this mutant would be an IGF independent effect [22]. On infection with Ad:EV, Ad:IGFBP-3 and Ad:IGFBP-3^GGG^ mutant, semi-quantitative PCR shows that the effect of Ad:IGFBP-3^GGG^ on the mRNA levels of GLUT4, IRS-1, adiponectin and MCP-1 was similar to Ad:IGFBP-3 suggesting that IGFBP-3 effect on the NF-κB pathway in adipocytes was IGF-independent (Figure 3C). Protein expression of IRS-1, GLUT4, adiponectin and MCP-1 confirmed that IGFBP-3 inhibits the NF-κB pathway and successfully restores the repressed levels of IRS-1, GLUT4 and adiponectin in ASCs in an IGF-independent manner (Figure 3D).

### TNF-α Induces Insulin Resistance in Human Adipocytes

The dose dependent effect of insulin on increasing glucose uptake in ASCs has been demonstrated in Figure 3E. However, visceral obesity is accompanied by release of various adipokines that leads to an inflammatory state resulting in insulin resistance wherein cells cease to respond to insulin presence. Figure 3F shows that increase in glucose uptake in the adipocytes on addition of insulin is inhibited in the presence of TNF-α, showing its role in insulin resistance. The IKK inhibitor treatment restores glucose uptake by the cells indicating that the decrease in glucose uptake by TNF-α was mediated via the NF-κB pathway.

### IGFBP-3 Negates TNF-α Induced Inhibition of Glucose Uptake in Human Adipocytes

In order to determine the effect of IGFBP-3 on TNF-α-induced insulin resistance, adipocytes were infected with IGFBP-3 alone and in the presence of TNF-α and insulin. Figure 3G shows that insulin alone increased the glucose uptake to almost 400 percent, however no change in glucose uptake was observed in the presence of IGFBP-3 alone, suggesting that IGFBP-3 under normal conditions does not regulate glucose metabolism. On the contrary, cells treated with TNF-α and insulin together showed more than a 50 percent decrease in glucose uptake but infection with IGFBP-3 abolished the TNF-α effect and restored levels of glucose uptake. Therefore, IGFBP-3 abrogates TNF-α-mediated inhibition of glucose uptake in human adipocytes and does not affect normal glucose metabolism.

### TNF-α Activates the NF-κB Pathway in HAECs

**Figure 4:**
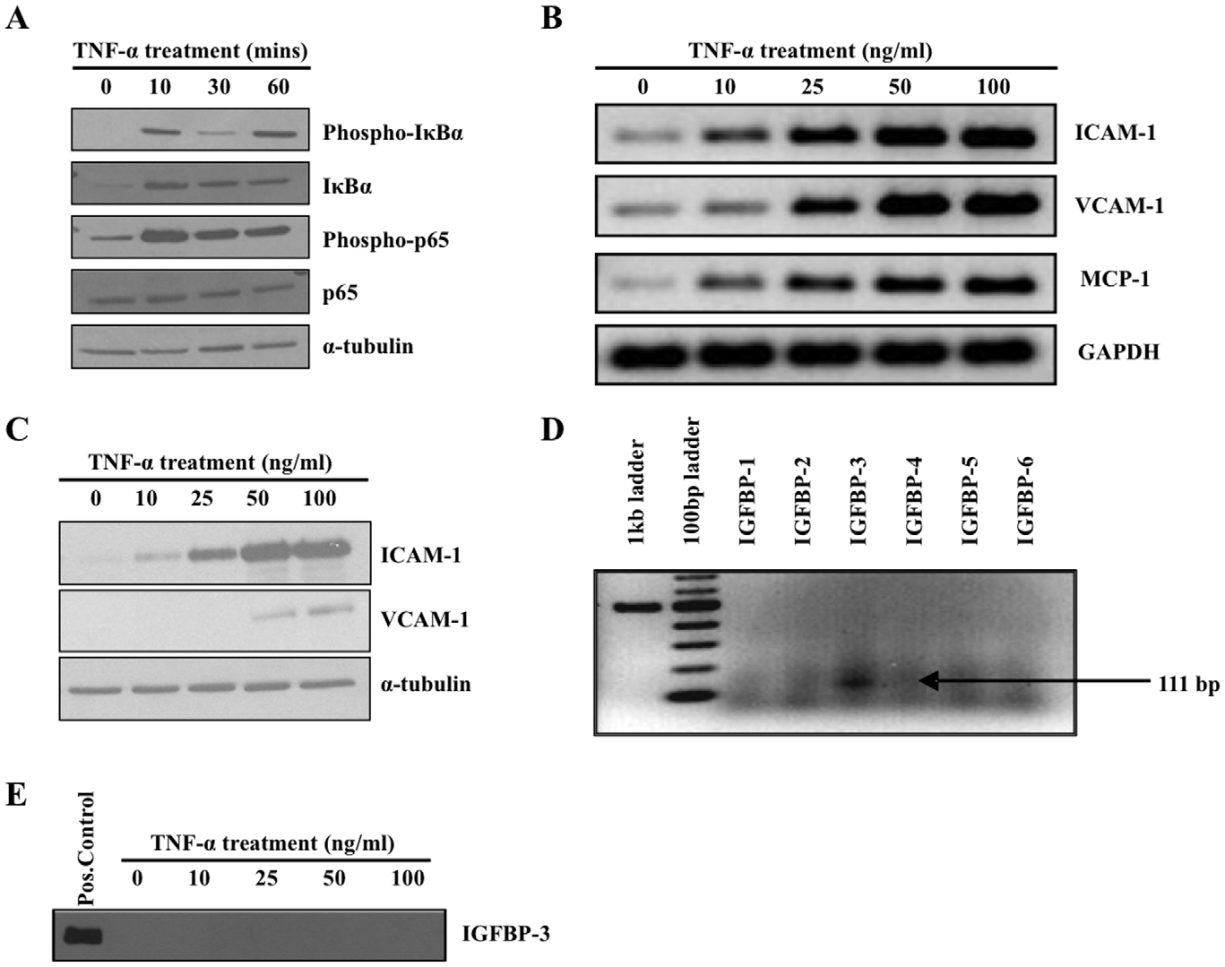
TNF-α activates NF-κB pathway in HAEC cells. (A) HAECs were treated with 50 ng/ml TNF-α and lysates were collected at 0, 10, 30 and 60 minutes. The cell lysates were analyzed by western blotting to observe the activation of NF-κB pathway. The figure shows phospho-IκBα and phospho-p65 confirming activation of NF-κB pathway by TNF-α in HAEC. (B) Dose dependent increase in the mRNA levels of ICAM-1, VCAM-1 and MCP-1 upon TNF-α treatment was analyzed by RT-PCR and GAPDH was the internal control for normalizing mRNA levels. (C) Dose dependent increase in protein levels of ICAM-1 and VCAM-1 by immunoblotting. α-tubulin was used as the loading control. (D) The expression profile of IGFBPs in HAECs was analyzed by RT-PCR using primers IGFBP-1 through IGFBP-6. PCR products were run on a 2% agarose gel. (E) HAEC cells were treated with TNF-α for 48 hours and subsequently analyzed for IGFBP-3 expression in conditioned media (CM). HAECs infected with Ad:IGFBP-3 was used a positive control.

In obesity, cytokines like TNF-α are elevated in circulation and result in cardiovascular endothelial dysfunction, plaque formation and CVD [23]. We studied the effect of TNF-α on cardiovascular endothelial cells by treating HAECs with TNF-α and observed a cyclic pattern in the levels of phospho-IκBα and total IκBα and an increase in phospho-p65 levels (Figure 4A). Figure 4B, shows that the downstream targets of the NF-κB pathway, ICAM-1, VCAM-1 and MCP-1 mRNA levels increased in a dose dependent manner ranging 0–100 ng/ml of TNF-α treatment and Figure 4C shows a dose dependent increase in the protein expression of ICAM-1 and VCAM-1 with TNF-α treatment.

### Expression Pattern of IGFBPs in HAECs

**Figure 5:**
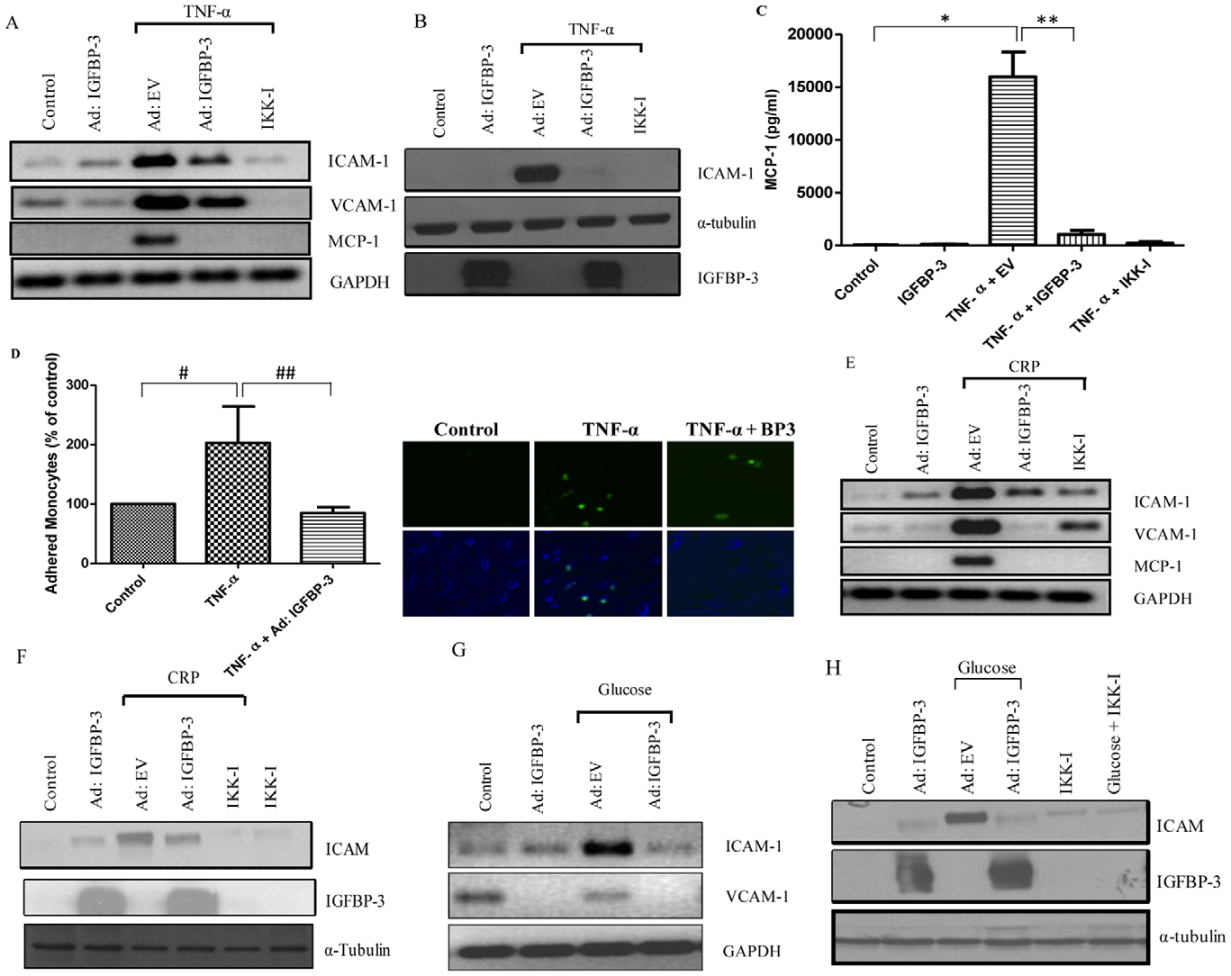
Effect of IGFBP-3 on NF-κB pathway in HAECs: Effect of IGFBP-3 on TNF-α induced NF-κB activation in HAEC cells. HAEC cells were infected with Ad:EV (m.o.i.100) or Ad:IGFBP-3 (m.o.i.100) for 24 hours followed by TNF-α treatment (50 ng/ml) for 24 hours and were analyzed for mRNA and protein expression by RT-PCR and western blotting respectively. Cells were also treated with 2.5 µM IKK-IV inhibitor. Figure (A) shows the mRNA levels for ICAM-1, VCAM-1 and MCP-1 for the treated samples and (B) shows protein expression for ICAM-1, IGFBP-3 and α-tubulin after IGFBP-3 infection by western blotting. IGFBP-3 blot confirms expression after adenovirus infection. HAECs were treated with IKK Inhibitor to confirm the involvement of the NF-κB pathway. The data demonstrates that IGFBP-3 inhibits TNF-α-induced NF-κB activity consequently inhibiting the mRNA and protein levels of ICAM-1, VCAM-1 and MCP-1. (C) A similar experiment was performed with IGFBP-3 infection and TNF-α treatment and MCP-1 levels were quantified in the CM using ELISA. n = 3, in duplicates; *, p<0.001;**, p<0.001. (D) HAEC cells were infected with Ad:IGFBP-3 and treated with 50 ng/ml of TNF-α. At the end of the incubation, monocyte cell adhesion assay was performed. 1*105 monocytes were stained with Calcein AM fluorescent dye and the stained cell suspension was layered over the HAEC cells. After 30 minutes of incubation non-adherent cells were removed and only the adhered cells were analyzed by fluorescent microscopy. The figure shows a representative field of each well. The number of adhered monocytes on the HAEC monolayer is depicted as a bar graph. n = 2, in triplicates; #, p<0.05;##, p<0.01. Effect of IGFBP-3 on CRP- and glucose-induced NF-κB activation in HAEC cells: HAEC cells were infected with adenovirus constructs for control and IGFBP-3. Simultaneously, cells were treated with 50 ng/ml CRP for 6 hrs or 30 mM of glucose for five days respectively. Cells were analyzed for effect on the mRNA levels (E, G) and protein expression (F, H) respectively after CRP or glucose treatment.

Figure 4D shows detectable endogenous levels of IGFBP-3 mRNA; however levels of other IGFBPs were not detected. Furthermore, Figure 5E shows no detectable levels of IGFBP-3 at the protein level and TNF-α treatment alone does not alter the levels of IGFBP-3 protein secreted in the conditioned media.

### IGFBP-3 Inhibits TNF-α Induced Activation of NF-κB Activity in HAECs

To determine if IGFBP-3 also plays an anti-inflammatory role in HAECs, TNF-α treated HAECs were infected with Ad:IGFBP-3 and the mRNA and expression levels of NF-κB-regulated target genes were analyzed. Figure 5A shows that IGFBP-3 alone has no effect on the mRNA levels however, infection with Ad:IGFBP-3, in the presence of TNF-α (50 ng/ml) significantly downregulates the mRNA levels of TNF-α-induced ICAM-1, VCAM-1 and MCP-1. IKK inhibitor treatment confirms that TNF-α and IGFBP-3 mediate their effects by regulating the NF-κB pathway. Figure 5B shows the protein profile for ICAM-1, confirming that IGFBP-3 inhibits TNF-α induced NF-κB activity in HAECs.

### IGFBP-3 Suppresses MCP-1 Levels and Inhibits Monocyte Adhesion to HAEC Monolayer

One of the consequences of chronic inflammatory state induced by the adipokines secreted by the visceral adipose tissue is increase in the recruitment of monocytes to cardiovascular endothelial cells, eventually leading to plaque formation. *In vitro* data shows that TNF-α-treated HAECs show increase in MCP-1 levels, whereas IGFBP-3 abolishes TNF-α effects (Figure 5C). Since MCP-1 levels are indicative of monocyte adhesion in cells, we performed a monocyte adhesion experiment that showed that HAECs treated with TNF-α lead to a two-fold increase in monocyte adhesion to a monolayer of heart cells and infection with Ad:IGFBP-3 inhibited monocyte adhesion to HAEC cells (Figure 5D). This confirms that IGFBP-3 inhibits a biological effect of TNF-α-induced pro-inflammatory activity in HAECs.

### IGFBP-3 Inhibits the Action of CVD Risk Factors, CRP and High Glucose

Other factors in circulation that adversely contribute towards CVD include insulin resistance-induced high glucose and CRP. Increase in the mRNA levels of ICAM-1, VCAM-1 and MCP-1 was observed on addition of CRP (Figure 5E) or high glucose (Figure 5G) and infection with IGFBP-3 significantly decreased these levels. We also confirm that both CRP (Figure 5F) and high glucose (Figure 5H) have a similar effect even on the protein levels of the NF-κB downstream targets and HAEC cells infected with Ad:IGFBP-3 reduce these levels.

### IGFBP-3 Inhibits the NF-κB Pathway in an IGF-independent Manner

**Figure 6:**
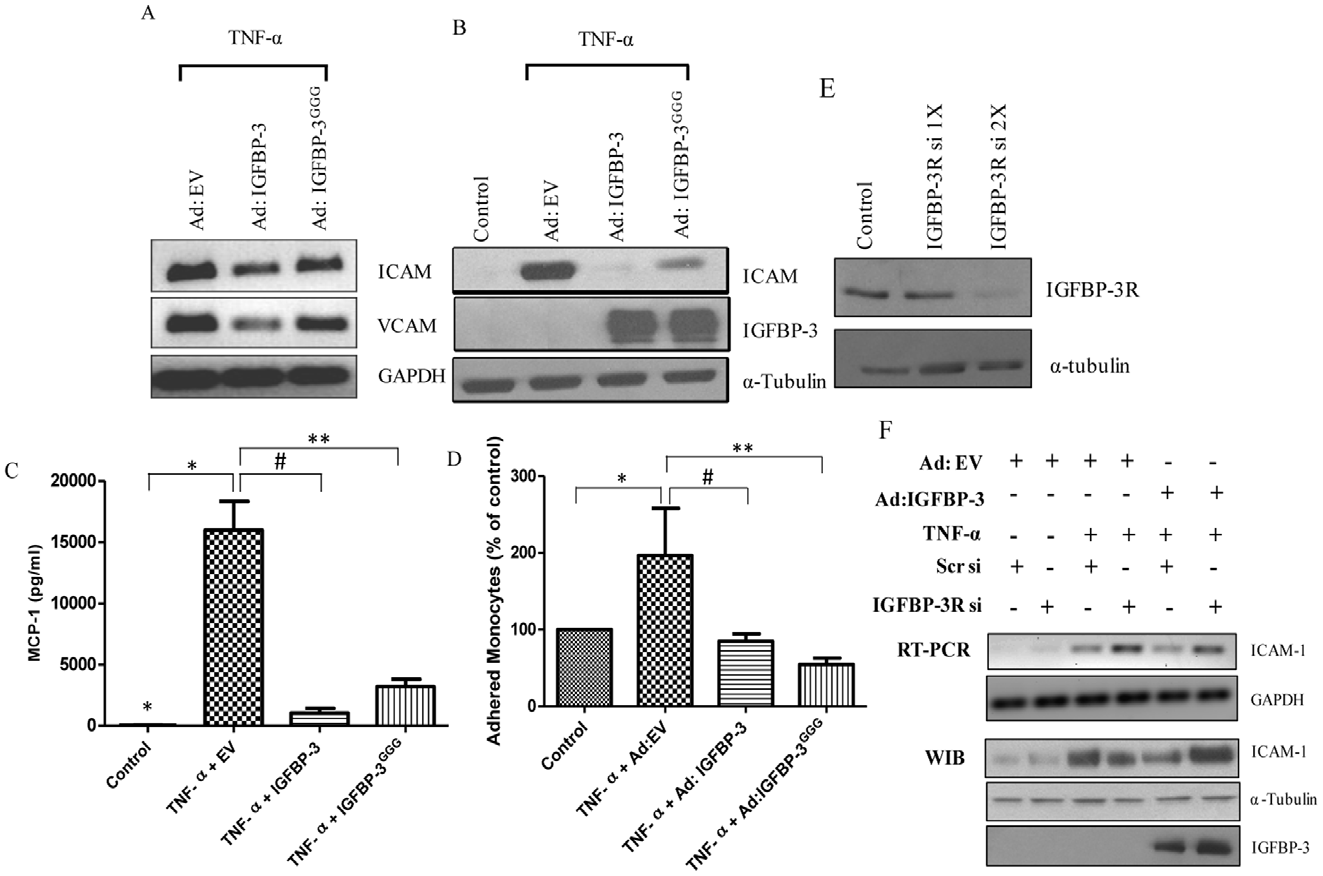
IGFBP-3 inhibits NF-κB activity in an IGF-independent manner in HAECs. HAEC cells were infected with Ad:EV (m.o.i.100) or Ad:IGFBP-3 (m.o.i.100) and Ad:IGFBP-3GGG (m.o.i. 100) followed by TNF-α treatment for 24 hours and were analyzed for mRNA (A) and protein expression (B) by RT-PCR and western blotting respectively. (C) A similar experiment was performed with infection with Ad:EV, Ad:IGFBP-3 or Ad:IGFBP-3GGG and TNF-α treatment and MCP-1 levels were quantified in the CM using ELISA. n = 2, in triplicates; *,#,**, p<0.001 (D) HAEC cells were infected with Ad:IGFBP-3 or Ad:IGFB3GGG and treated with 50 ng/ml of TNF-α. At the end of the incubation period, monocyte cell adhesion assay was performed. The numbers of adhered monocytes on the HAEC monolayer are depicted as a bar graph. n = 2 in triplicates; *, p = 0.05; #, **, p<0.05. (E) HAEC cells were transfected with siRNA against IGFBP-3R once or two consecutive times and cells were harvested to determine the knockdown of IGFBP-3R by western blotting. HAEC cells were transfected with either scrambled siRNA or siRNA against IGFBP-3R. (F) The cells were then infected with Ad:EV (m.o.i.100) or Ad:IGFBP-3 (m.o.i.100) followed by TNF-α treatment for 24 hours and were analyzed for mRNA and protein expression.

In order to elucidate the underlying mechanism in the inhibition of NF-κB pathway by IGFBP-3, and to determine if this effect was an IGF-independent, HAECs were infected with Ad:IGFBP-3^GGG^ and then treated with TNF-α. Figure 6A shows that infection with Ad: IGFBP-3^GGG^ followed by TNF-α treatment inhibits the mRNA levels of ICAM-1 and VCAM-1 similar to Ad:IGFBP-3 compared to the control. This pattern was also seen in protein expression (Figure 6B). An MCP-1 ELISA assay determined that both Ad:IGFBP-3^GGG^ and Ad:IGFBP-3 inhibited TNF-α- induced MCP-1 levels (Figure 6C). Ad:IGFBP-3^GGG^ also inhibited monocyte adhesion to the HAECs comparable to the effect of Ad:IGFBP-3 (Figure 6D). Furthermore, to determine if IGFBP-3 inhibits the NF-κB pathway and its downstream targets, through IGFBP-3R, HAECs were transfected with siRNA to knockdown endogenous levels of IGFBP-3R (Figure 6E). Figure 6F shows that Ad:IGFBP-3 inhibited expression of ICAM-1 by 40 percent and knockdown of IGFBP-3R negates the inhibitory effect of IGFBP-3 on TNF-α-induced levels of downstream target of the NF-κB pathway and restores the levels of ICAM-1.

### Differential Expression of IGFBP-3 in Classically and Alternately Activated Macrophages

**Figure 7:**
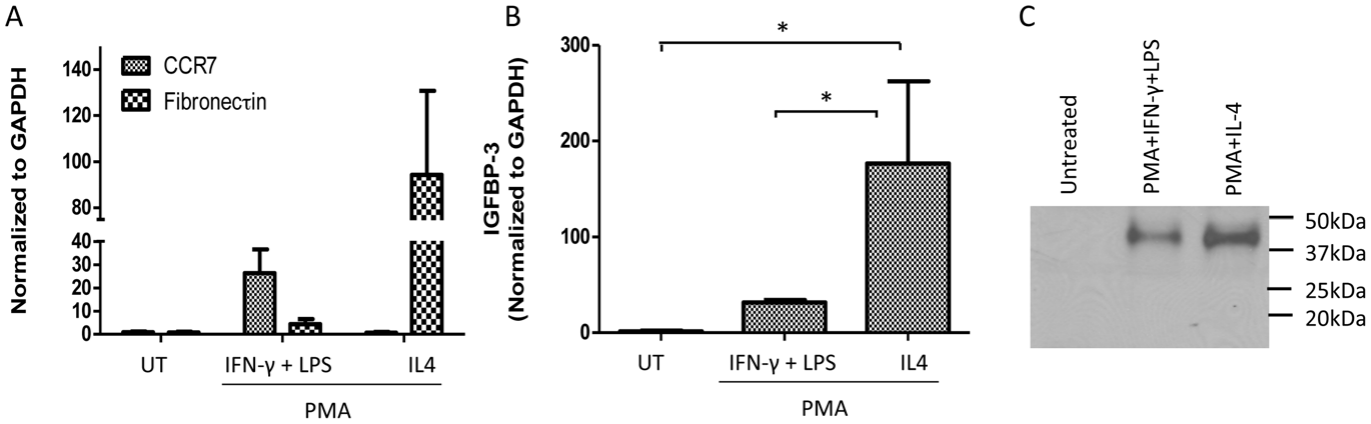
Differential expression of IGFBP-3 in classically and alternately activated macrophages. PMA treated macrophages were differentiated into M1 and M2 macrophages by treating them with IFN-γ+LPS and IL-4 respectively. Figure (A) shows that M1 shows increased levels of CCR7 (M1 marker) mRNA and Fibronectin (M2 marker) levels are higher in M2 when compared to Untreated (B) The bar graph below shows that mRNA levels of IGFBP-3 are higher in M2 when compare to both untreated and M1 differentiated macrophages, * p<0.05. For protein analysis, equal numbers of THP-1 cells were plated and differentiated to M1 and M2 as mentioned above and IGFBP-3 protein levels were analyzed on a western blot by loading equal volumes of conditioned media (C).

In addition to adipocytes, tissue macrophages have also been shown to be responsible for maintaining an inflammatory response in adipose tissue [24]. Based on specific environmental stimuli, macrophage activation can be categorized into two responses: classical (M1) or alternative (M2) macrophages [25]. It has been shown that obesity induces a phenotypic switch from an anti-inflammatory M2 state to a pro-inflammatory M1 state [26]. To determine if M1 and M2 activation affects the levels of IGFBP-3, we used human monocytes (THP-1 cells) and differentiated them to M1 and M2 macrophages using phorbol-12-myristate-13-acetate (PMA)+IFN-γ+LPS and PMA+IL4 respectively. We used CCR-7 and fibronectin as markers for M1 and M2 to confirm that our treatments did differentiate the monocytes to M1 and M2 macrophages respectively [27]. Monocytes treated with PMA+IFN-γ+LPS show higher levels of CCR7 (M1 marker) and those treated with PMA+IL4 show increased levels of fibronectin (M2 marker) (Figure 7A). On confirming macrophage differentiation, we analyzed the mRNA and protein levels of IGFBP-3. Figure 7B shows that M2 macrophages express approximately 5.5 fold higher levels of IGFBP-3 mRNA when compared to M1 macrophages. Further protein analysis of IGFBP-3 in the conditioned media reiterates that M2 macrophages express higher levels of IGFBP-3 than M1 macrophages without any detectable proteolyzed fragments (Figure 7C). This result suggests that in addition to reduced levels of biologically active, intact IGFBP-3 in circulation in obese state due to increased proteolysis, another possible mechanism for reduced anti-inflammatory function of IGFBP-3 in the adipose tissue could be due to reduction in the presence of anti-inflammatory M2 macrophages resulting in reduced expression of IGFBP-3.

## Discussion

Visceral fat obesity and not subcutaneous fat obesity, correlates significantly with insulin resistance [28], hypertension [29] and cardiac dysfunction [30]. In obesity, fat depots increase the levels of cytokines that act in a paracrine manner further entering circulation and resulting in a state of systemic inflammation [31].

In addition to its ability to modulate IGF availability, IGFBP-3 has also been shown to have IGF-independent effects [10]. IGFBP-3 has been shown to be associated with CVD [7], obesity [8] and insulin resistance [32]. We and others have shown that IGFBP-3 acts as an anti-inflammatory factor, capable of inhibiting the NF-κB pathway [21], [22], [33]. Reports suggest that IGFBP-3 may act as an insulin antagonist. IGFBP-3 has been shown to inhibit insulin-stimulated glucose uptake in adipocytes, human omental adipose tissue, and in Sprague-Dawley rats [34], [35]. It is of note that these studies characterize IGFBP-3 function in a normal physiological setting. However, we, in our study show that IGFBP-3 may have a distinct function in pathologic, disease states. Nevertheless, our data demonstrate that IGFBP-3 does not affect insulin-induced glucose uptake under normal conditions (Figure 3G) A recent study shows that diet-induced obese rats show a downregulation of IGFBP-3 expression in mesenteric fat when compared to their lean counterparts, suggesting that IGFBP-3 may have a protective effect against obesity [36].

IGF-1 has been shown to increase insulin sensitivity and improve glycemic control in patients with type 2 diabetes [37]. In our data, we see that insulin resistance exists in both overweight and obese adolescents, in spite of the levels of IGF-1 increasing in the overweight and obese individuals. In some instances, it has been shown that at low concentrations, IGF-1 stimulates proliferation of arterial smooth muscle cells and at high concentrations it inhibits smooth muscle cell proliferation. This suggests that IGF-1 could be both a pro-atherogenic and an anti-atherogenic factor [38] indicating that IGFBP-3 levels and its regulation on IGF-1 may be a better indicator and marker of insulin resistance.

Proteolytic degradation of IGFBP-3 has been reported in several catabolic conditions and proteases found in a variety of biological fluids can degrade IGFBP-1-6 into fragments that greatly reduce affinity for IGF-I and IGF-II, thereby increasing the concentration of free IGF [39]. Therefore, IGFBP proteolysis directly regulates IGF receptor signaling [40]. However, it is not known if the proteolyzed IGFBP-3 fragments still possess intrinsic biological functions of intact IGFBP-3 such as IGF-independent anti-inflammatory function of IGFBP-3. It is of note that the mid region of IGFBP-3 molecule possesses an IGFBP-3R binding domain [13] whereas IGFBP-3 proteases appear to cleave the IGFBP-3 mid region [41], [42]. Taken together, these findings strongly suggest that IGFBP-3 proteolysis may result in loss of IGFBP-3R binding ability and subsequent its anti-inflammatory function. In this study we show that IGFBP-3 proteolysis was significantly increased in overweight as well as obesity groups compared to normal controls by both western blotting and protease activity assay. Its correlation with HOMA-insulin resistance suggests that proteolysis of IGFBP-3 may be involved in the pathogenesis of obesity-induced insulin resistance. Though there are conflicting reports about the effect of obesity on IGFBP-3 proteolysis [43] it is evident that IGFBP-3 proteolysis is increased in patients with non insulin-dependent diabetes mellitus [17]. This further raises an interesting concept that IGFBP-3 proteolysis may result in decrease of intact IGFBP-3 in circulation and the local environment, thereby suppressing the intrinsic anti-inflammatory function of IGFBP-3. Also, studies show that adipose tissue of a lean mice have a macrophage content of less than 10% of the total cell nuclei count, whereas obese mice present more than 50% of macrophage content [24], [44]. It has been shown that obesity induces a phenotypic switch from an anti-inflammatory M2 state to a pro-inflammatory M1 state [26]. On the other hand, Lumeng *et al* in his study suggests that during obesity, adipose tissue macrophages are newly recruited from monocyte precursors and these macrophages display M1 characteristics [45]. M2 macrophages are known to maintain glucose tolerance, insulin sensitivity and general adipocyte function that can prevent development of obesity due to diet and type-2 diabetes [25], [45]. In both humans and mice, lipidystrophy has been associated with overexpression of the M1 phenotype of macrophages [46], [47]. In accordance with the role of macrophages and the anti-inflammatory properties of IGFBP-3, in our study we show that M2 macrophages express higher levels of IGFBP-3 than M1, suggesting that decreased expression of IGFBP-3 by macrophages in addition to occurrence of IGFBP-3 proteolysis in circulation may contribute towards obesity and insulin resistance.

Visceral adipocytes are highly responsive to TNF-α mediated activation of NF-κB pathway [18]. Our study shows that IGFBP-3 inhibits TNF-α-induced NF-κB activity in differentiated human adipocytes, inhibiting TNF-α-mediated deregulation of insulin signaling in ASCs. IGFBP-3 restores the levels of IRS-1, GLUT4 and adiponectin and inhibits MCP-1 levels. NF-κB pathway has been shown to be active in human atherosclerotic plaques and in contrast, healthy vessels are devoid of such increased activity. Presence of activated NF-κB pathway is observed in smooth muscle cells, endothelial cells of atherosclerotic lesions [48] and in the intimal cells of the coronary arteries of pigs that are fed a hypercholesterolemic diet [49]. Many genes regulated by the NF-κB pathway such as ICAM-1, VCAM-1, IL-8 and MCP-1 help in the recruitment of monocytes to the arterial intima. This further leads to the migration and proliferation of smooth muscle cells resulting in plaque formation and atherosclerosis. Since pharmacological inhibition or genetic knockdown of adhesion molecules, MCP-1 and TNF-α is known to retard the progression of CVD in atherosclerotic models [50], [51], in our study we investigated and determined that in HAECs, IGFBP-3 inhibits TNF-α-induced adhesion molecules; ICAM-1, VCAM-1 and MCP-1, downstream of the NF-κB pathway. In addition to inhibiting TNF-α-induced MCP-1 levels in the cells, IGFBP-3 also prevents adhesion of monocytes to HAECs. High glucose and CRP, that are pro-atherogenic and pro-inflammatory factors also activate the NF-κB pathway, upregulating adhesion proteins and recruiting monocytes to the vascular endothelium [52] and our data shows that IGFBP-3 also inhibits CRP- and high glucose- induced NF-κB pathway in HAECs.

Taken together, these *in vitro* data provide convincing evidence that IGFBP-3 negates obesity-induced NF-κB activity in adipocytes and HAECs. Our data with IGFBP-3^GGG^ mutant further confirms that this inhibition of the NF-κB activity occurs in an IGF-independent manner. IGFBP-3R [13], is involved in the inhibition of TNF-α induced NF-κB signaling cascade via the activation of caspases and eventual apoptosis in cancer cells [11]. IGFBP-3 also inhibits NF-κB signaling in normal lung epithelial cells via activation of the IGFBP-3R that involves activation of caspase pathway and degradation of IκBα and p65-NF-κB proteins, thereby inhibiting TNF-α-induced activation of NF-κB signaling cascades and blocking the physiological manifestations of asthma and bronchial inflammation [12]. Not surprisingly, knockdown of endogenous IGFBP-3R in our *in vitro* heart cell system negates the biological effect of IGFBP-3 and restores the levels of ICAM-1, thereby suggesting that IGFBP-3 inhibits the NF-κB pathway via an IGFBP-3/IGFBP-3R axis.

In conclusion, these new discoveries for the inhibitory role of IGFBP-3 in obesity-induced insulin resistance and in the events occurring in the early stages of atherosclerosis, set the stage for a potential therapeutic role of IGFBP-3 in various aspects of metabolic syndrome.

## Materials and Methods

### Subjects

The study consisted of 197 subjects aged 12 to 13 years old among adolescents who visited the National Medical Center, Korea for medical examinations. The BMI percentiles for age and gender according to 2007 Korea Growth Charts for assessment of obesity were utilized. At the time of study enrollment no subject had either a chronic disease or acute illness, was taking medications for any condition, or had a history of smoking. The Hospital Ethnics Committee of the Severance Children’s hospital reviewed and approved the study, and written informed consent was obtained from guardians and study subjects.

### Anthropometric Measurements

Weight, height, BP and waist circumference were measured as previously described [24], [29].

### Blood Samples Analyses

All subjects were fasted for more than 12 hours. Whole blood samples were obtained by venipuncture. Sera were used for biochemical and hormonal assay within 8 hours and immunoblot assay was performed.

### Biochemical and Hormonal Analyses

Level of fasting serum glucose, insulin, total cholesterol, triglycerides, AST, ALT and HDL cholesterol were measured. Levels of LDL cholesterol were calculated as follows: LDL-cholesterol = total cholesterol – HDL-cholesterol–(triglyceride/5). CRP concentrations were measured by IMMAGE assay (Beckman Coulter, CA, USA), and fasting insulin levels were measured by electroluminescence immunoassay. HOMA-IR was calculated as follows: HOMA-IR = [fasting insulin (µIU/mL) x fasting glucose (mg/dL)/18]/22.5 [25].

### IGFBP-3 Protease Activity Assay

Recombinant human glycosylated IGFBP-3 was biotinylated by Biotin-XX microscale protein labeling kit (Invitrogen). Two ul sera and 5 ul biotinylated IGFBP-3 were incubated at 37°c for 15 minutes and subjected to WIB. IGFBP-3 protease activity was calculated from WIB as the sum of density of the fragment bands at 29 and 18 kDa divided by the sum of all fragments and intact IGFBP-3 at approximately 43, 29, and 18 kDa.

### ELISA Assays for IGF-I, IGFBP-3 and MCP-1 ELISA Assays

Human IGF-I ELISA (ALPCO,NH, USA), total IGFBP-3 (MEDIAGNOST, Reutlingen, Germany) and MCP-1 ELISA kits (BD Biosciences) were purchased and assays were performed according to manufacturer’s protocol.

### Reagents

Isobutyl-methylxanthine, dexamethasone, indomethacin, insulin glucose, TNF-α were purchased from Sigma, IKK inhibitor and CRP were obtained from Calbiochem, whereas Calcein AM from Molecular probes.

### Semi Quantitative and Quantitative PCR Analysis

Total RNA was extracted and reverse transcripted, and RTQ-PCR was performed with designed primers (Table 4) [18].

**Table 4:** Primer Sequences.

Genes	Primer sequence
IRS-1 fwd	5′-CCTGGATTTGGTCAAGGACT-3′
IRS-1 rev	5′-TCATTCTGCTGTGATGTCCA-3′
Glut4 fwd	5′- TTATTCGACCAGCATCTTCG-3′
Glut4 rev	5′-AGCAGAGCCACAGTCATCAG-3′
Adiponectin fwd	5′-TGCTGGGAGCTGTTCTACTG-3′
Adiponectin rev	5′-GTTTCACCGATGTCTCCCTT-3′
MCP-1 fwd	5′ - ATCAATGCCCCAGTCACC - 3′
MCP-1 rev	5′-AGTCTTCGGAGTTTGGG-3′
hβ2M fwd	5′- GTGCTCGCGCTACTCTCTCT-3′
hβ2M rev	5′- TCAATGTCGGATGGATGAAA-3′
IGFBP-3R fwd	5′- AGACAGGAACAAGACCCGGACATT -3′
IGFBP-3R rev	5′- ATAGGCAGGTTCCTGCAGTCCTTT-3′
ICAM-1 fwd	5′-CTGCAGACAGTGACCATC-3′
ICAM-1 rev	5′- GTCCAGTTTCCCGGACAA-3′
VCAM-1 fwd	5′- AGATGGCGCCTATACCATCCGAAA -3′
VCAM-1 rev	5′- AGAGCACGAGAAGCTCAGGAGAAA -3′
IGFBP-1 fwd	5′- AGGCTCTCCATGTCACCAACATCA -3′
IGFBP-1 rev	5′- TGTCTCCTGTGCCTTGGCTAAACT –3′
IGFBP-2 fwd	5′- GCATGGCCTGTACAACCTCAAACA-3′
IGFBP-2 rev	5′- AGCCTCCTGCTGCTCATTGTAGA -3′
IGFBP-3 fwd	5′- CAGAGCACAGATACCCAGAACTTC-3′
IGFBP-3 rev	5′-CACATTGAGGAACTTCAGGTGATT-3′
IGFBP-4 fwd	5′- TGCAGAAGCACTTCGCCAAA-3′
IGFBP-4, rev	5′- ATGATGTAGAGGTCCTCGTGGGTG-3′
IGFBP-5 fwd	5′- AAAGAGCTACCGCGAGCAAGTCAA -3′
IGFBP-5 rev	5′- ACAAACTTGGACTGGGTCAGCTTC-3′
IGFBP-6 fwd	5′- AACCGCAGAGACCAACAGAGGAAT-3′
IGFBP-6 rev	5′- TGGTCACAATTGGGCACGTAGAGT -3′
GAPDH fwd	5′- CCAATAGGCGCTCACTGTTCT -3′
GAPDH rev	5′- GCGAACTCACCCGTTGACT -3′

### Adenoviral Constructs

Ad:IGFBP-3 and Ad:IGFBP-3^GGG^ were generated and characterized as previously described [13].

### Growth, Differentiation and Maintenance of ASCs

Cells were grown to 100% confluence in the growth medium supplemented with 10% FBS. Confluent cells were incubated in DM for 6 days (change medium every 3 days), 1 day in MM and then switched to DM again. This cycle was repeated for a period of 21 days until cells were differentiated.

### Macrophage Differentiation

Cells were treated with 20 ng/ml PMA for 8 hours followed by treatment with 20 ng/ml of IFN-γ and 20 ng/ml LPS for differentiating them into M1 macrophages and 20 ng/ml IL-4 for M2 macrophages. The cells were then harvested for mRNA analysis after 20 hours and cell lysates and conditioned media were collected at 72 hours.

### Monocyte Adhesion Assay

Equal numbers of HAEC cells were plated, followed by adenovirus infection with Ad: EV, Ad: IGFBP-3 or Ad: IGFBP-3^GGG^ as required followed by TNF-α treatment with in respective wells. A fixed number of monocytes (THP-1) were stained with 2 µM Calcein AM and the adhered monocytes were counted using a fluorescence microscope for three representative fields.

### Glucose Uptake Assay

Differentiated adipocytes were cultured in serum free low glucose medium for 2 hours. In experiments involving adenovirus infection, two hours after infection cells were treated with 20 ng/ml of TNF-α. After 24 hours of incubation, cells were incubated with or without insulin in KRPH buffer for 20 min and subjected to glucose uptake assay.

### siRNA Transfection

Cells were transfected with siRNAs against IGFBP-3R (siGENOME SMARTpool, Dharmacon) as described previously [23].

### Statistical Analyses

To determine the statistical differences in clinical characteristics among the three groups, we used one-way analysis of variance (ANOVA). Pearson’s correlation coefficients were calculated to evaluate the relationship between the degree of IGFBP-3 proteolysis and clinical features. Results were considered significant when *P*<0.05. All statistical analyses were performed with SAS 9.13 service pack3 (SAS Institute, Cary, NC, USA). Statistical analyses for *in vitro* experiments in differentiated adipocytes and HAECs were performed by t-test.

## References

[1] Ogden CL, Carroll MD, Curtin LR, Lamb MM, Flegal KM (2010) Prevalence of high body mass index in US children and adolescents, 2007–2008. JAMA 303: 242–249. 20071470 10.1001/jama.2009.2012

[2] Dubuc PU (1976) The development of obesity, hyperinsulinemia, and hyperglycemia in ob/ob mice. Metabolism 25: 1567–1574. 994838 10.1016/0026-0495(76)90109-8

[3] Ouchi N, Kihara S, Funahashi T, Nakamura T, Nishida M, et al (2003) Reciprocal association of C-reactive protein with adiponectin in blood stream and adipose tissue. Circulation 107: 671–674. 12578865 10.1161/01.cir.0000055188.83694.b3

[4] Neels JG, Olefsky JM (2006) Inflamed fat: what starts the fire? J Clin Invest 116: 33–35. 16395402 10.1172/JCI27280 PMC1323268

[5] Hwa V, Oh Y, Rosenfeld RG (1999) The insulin-like growth factor-binding protein (IGFBP) superfamily. Endocr Rev 20: 761–787. 10605625 10.1210/edrv.20.6.0382

[6] Kaplan RC, McGinn AP, Pollak MN, Kuller LH, Strickler HD, et al (2007) Association of total insulin-like growth factor-I, insulin-like growth factor binding protein-1 (IGFBP-1), and IGFBP-3 levels with incident coronary events and ischemic stroke. J Clin Endocrinol Metab 92: 1319–1325. 17264182 10.1210/jc.2006-1631

[7] Spilcke-Liss E, Friedrich N, Dorr M, Schminke U, Volzke H, et al. (2011) Serum Insulin-like Growth Factor-I and its Binding Protein 3 in their Relation to Intima Media Thickness: Results of the Study of Health in Pomerania (SHIP). Clin Endocrinol (Oxf). 10.1111/j.1365-2265.2011.04010.x 21521279

[8] Gomez JM, Maravall FJ, Gomez N, Navarro MA, Casamitjana R, et al (2004) The IGF-I system component concentrations that decrease with ageing are lower in obesity in relationship to body mass index and body fat. Growth Horm IGF Res 14: 91–96. 15123168 10.1016/j.ghir.2003.11.004

[9] Kielczewski JL, Hu P, Shaw LC, Li Calzi S, Mames RN, et al (2011) Novel protective properties of IGFBP-3 result in enhanced pericyte ensheathment, reduced microglial activation, increased microglial apoptosis, and neuronal protection after ischemic retinal injury. Am J Pathol 178: 1517–1528. 21435441 10.1016/j.ajpath.2010.12.031 PMC3078432

[10] Oh Y, Muller HL, Lamson G, Rosenfeld RG (1993) Insulin-like growth factor (IGF)-independent action of IGF-binding protein-3 in Hs578T human breast cancer cells. Cell surface binding and growth inhibition. J Biol Chem 268: 14964–14971. 7686909

[11] Han J, Jogie-Brahim S, Harada A, Oh Y (2011) Insulin-like growth factor-binding protein-3 suppresses tumor growth via activation of caspase-dependent apoptosis and cross-talk with NF-kappaB signaling. Cancer Lett 307: 200–210. 21536375 10.1016/j.canlet.2011.04.004

[12] Lee YC, Jogie-Brahim S, Lee DY, Han J, Harada A, et al (2011) Insulin-like growth factor-binding protein-3 (IGFBP-3) blocks the effects of asthma by negatively regulating NF-kappaB signaling through IGFBP-3R-mediated activation of caspases. J Biol Chem 286: 17898–17909. 21383009 10.1074/jbc.M111.231035 PMC3093865

[13] Ingermann AR, Yang YF, Han J, Mikami A, Garza AE, et al (2010) Identification of a novel cell death receptor mediating IGFBP-3-induced anti-tumor effects in breast and prostate cancer. J Biol Chem 285: 30233–30246. 20353938 10.1074/jbc.M110.122226 PMC2943278

[14] Hossenlopp P, Segovia B, Lassarre C, Roghani M, Bredon M, et al (1990) Evidence of enzymatic degradation of insulin-like growth factor-binding proteins in the 150K complex during pregnancy. J Clin Endocrinol Metab 71: 797–805. 1698199 10.1210/jcem-71-4-797

[15] AM HZ, Collard TJ, Malik K, Hicks DJ, Paraskeva C, et al (2006) Induction of apoptosis by the 16-kDa amino-terminal fragment of the insulin-like growth factor binding protein 3 in human colonic carcinoma cells. Int J Oncol 29: 1279–1286. 17016662

[16] Jogie-Brahim S, Feldman D, Oh Y (2009) Unraveling insulin-like growth factor binding protein-3 actions in human disease. Endocr Rev 30: 417–437. 19477944 10.1210/er.2008-0028 PMC2819737

[17] Bang P, Brismar K, Rosenfeld RG (1994) Increased proteolysis of insulin-like growth factor-binding protein-3 (IGFBP-3) in noninsulin-dependent diabetes mellitus serum, with elevation of a 29-kilodalton (kDa) glycosylated IGFBP-3 fragment contained in the approximately 130- to 150-kDa ternary complex. J Clin Endocrinol Metab 78: 1119–1127. 7513716 10.1210/jcem.78.5.7513716

[18] Maury E, Noel L, Detry R, Brichard SM (2009) In vitro hyperresponsiveness to tumor necrosis factor-alpha contributes to adipokine dysregulation in omental adipocytes of obese subjects. J Clin Endocrinol Metab 94: 1393–1400. 19174496 10.1210/jc.2008-2196

[19] Skolnik EY, Marcusohn J (1996) Inhibition of insulin receptor signaling by TNF: potential role in obesity and non-insulin-dependent diabetes mellitus. Cytokine Growth Factor Rev 7: 161–173. 8899294 10.1016/1359-6101(96)00021-4

[20] Olson AL, Pessin JE (1996) Structure, function, and regulation of the mammalian facilitative glucose transporter gene family. Annu Rev Nutr 16: 235–256. 8839927 10.1146/annurev.nu.16.070196.001315

[21] Matsuzawa Y (2010) Adiponectin: a key player in obesity related disorders. Curr Pharm Des 16: 1896–1901. 20370675 10.2174/138161210791208893

[22] Buckway CK, Wilson EM, Ahlsen M, Bang P, Oh Y, et al (2001) Mutation of three critical amino acids of the N-terminal domain of IGF-binding protein-3 essential for high affinity IGF binding. J Clin Endocrinol Metab 86: 4943–4950. 11600567 10.1210/jcem.86.10.7936

[23] Ouwens DM, Sell H, Greulich S, Eckel J (2010) The role of epicardial and perivascular adipose tissue in the pathophysiology of cardiovascular disease. J Cell Mol Med 14: 2223–2234. 20716126 10.1111/j.1582-4934.2010.01141.x PMC3822561

[24] Weisberg SP, McCann D, Desai M, Rosenbaum M, Leibel RL, et al (2003) Obesity is associated with macrophage accumulation in adipose tissue. J Clin Invest 112: 1796–1808. 14679176 10.1172/JCI19246 PMC296995

[25] Odegaard JI, Chawla A (2011) Alternative macrophage activation and metabolism. Annu Rev Pathol 6: 275–297. 21034223 10.1146/annurev-pathol-011110-130138 PMC3381938

[26] Dalmas E, Clement K, Guerre-Millo M (2011) Defining macrophage phenotype and function in adipose tissue. Trends Immunol 32: 307–314. 21616718 10.1016/j.it.2011.04.008

[27] Martinez FO, Gordon S, Locati M, Mantovani A (2006) Transcriptional profiling of the human monocyte-to-macrophage differentiation and polarization: new molecules and patterns of gene expression. J Immunol 177: 7303–7311. 17082649 10.4049/jimmunol.177.10.7303

[28] Yamashita S, Nakamura T, Shimomura I, Nishida M, Yoshida S, et al (1996) Insulin resistance and body fat distribution. Diabetes Care 19: 287–291. 8742584 10.2337/diacare.19.3.287

[29] Kanai H, Matsuzawa Y, Kotani K, Keno Y, Kobatake T, et al (1990) Close correlation of intra-abdominal fat accumulation to hypertension in obese women. Hypertension 16: 484–490. 2228147 10.1161/01.hyp.16.5.484

[30] Nakamura T, Tokunaga K, Shimomura I, Nishida M, Yoshida S, et al (1994) Contribution of visceral fat accumulation to the development of coronary artery disease in non-obese men. Atherosclerosis 107: 239–246. 7980698 10.1016/0021-9150(94)90025-6

[31] Guzik TJ, Mangalat D, Korbut R (2006) Adipocytokines - novel link between inflammation and vascular function? J Physiol Pharmacol 57: 505–528. 17229978

[32] Kim HS, Ali O, Shim M, Lee KW, Vuguin P, et al (2007) Insulin-like growth factor binding protein-3 induces insulin resistance in adipocytes in vitro and in rats in vivo. Pediatr Res 61: 159–164. 17237715 10.1203/pdr.0b013e31802d8a30

[33] Williams AC, Smartt H, AM HZ, Macfarlane M, Paraskeva C, et al (2007) Insulin-like growth factor binding protein 3 (IGFBP-3) potentiates TRAIL-induced apoptosis of human colorectal carcinoma cells through inhibition of NF-kappaB. Cell Death Differ 14: 137–145. 16645643 10.1038/sj.cdd.4401919

[34] Silha JV, Gui Y, Murphy LJ (2002) Impaired glucose homeostasis in insulin-like growth factor-binding protein-3-transgenic mice. Am J Physiol Endocrinol Metab 283: E937–945. 12376320 10.1152/ajpendo.00014.2002

[35] Moses AC, Young SC, Morrow LA, O’Brien M, Clemmons DR (1996) Recombinant human insulin-like growth factor I increases insulin sensitivity and improves glycemic control in type II diabetes. Diabetes 45: 91–100. 8522066 10.2337/diab.45.1.91

[36] Palau N, Rebuffat SA, Altirriba J, Piquer S, Hanzu FA, et al (2012) Role of IGFBP-3 in the regulation of beta-cell mass during obesity: adipose tissue/beta-cell cross talk. Endocrinology 153: 177–187. 22067319 10.1210/en.2011-0181

[37] Gatenby VK, Kearney MT (2010) The role of IGF-1 resistance in obesity and type 2 diabetes-mellitus-related insulin resistance and vascular disease. Expert Opin Ther Targets 14: 1333–1342. 21058922 10.1517/14728222.2010.528930

[38] Schuler-Luttmann S, Monnig G, Enbergs A, Schulte H, Breithardt G, et al (2000) Insulin-like growth factor-binding protein-3 is associated with the presence and extent of coronary arteriosclerosis. Arterioscler Thromb Vasc Biol 20: E10–15. 10764692

[39] Maile LA, Holly JM (1999) Insulin-like growth factor binding protein (IGFBP) proteolysis: occurrence, identification, role and regulation. Growth Horm IGF Res 9: 85–95. 10.1054/ghir.1999.0096 10373341

[40] Bunn RC, Fowlkes JL (2003) Insulin-like growth factor binding protein proteolysis. Trends Endocrinol Metab 14: 176–181. 12714278 10.1016/s1043-2760(03)00049-3

[41] Bang P, Fielder PJ (1997) Human pregnancy serum contains at least two distinct proteolytic activities with the ability to degrade insulin-like growth factor binding protein-3. Endocrinology 138: 3912–3917. 9275081 10.1210/endo.138.9.5371

[42] Firth SM, Baxter RC (1995) The role of glycosylation in the action of IGFBP-3. Prog Growth Factor Res 6: 223–229. 8817665 10.1016/0955-2235(95)00009-7

[43] Ballerini MG, Ropelato MG, Domene HM, Pennisi P, Heinrich JJ, et al (2004) Differential impact of simple childhood obesity on the components of the growth hormone-insulin-like growth factor (IGF)-IGF binding proteins axis. J Pediatr Endocrinol Metab 17: 749–757. 15237710 10.1515/jpem.2004.17.5.749

[44] Xu H, Barnes GT, Yang Q, Tan G, Yang D, et al (2003) Chronic inflammation in fat plays a crucial role in the development of obesity-related insulin resistance. J Clin Invest 112: 1821–1830. 14679177 10.1172/JCI19451 PMC296998

[45] Lumeng CN, Deyoung SM, Bodzin JL, Saltiel AR (2007) Increased inflammatory properties of adipose tissue macrophages recruited during diet-induced obesity. Diabetes 56: 16–23. 17192460 10.2337/db06-1076

[46] Odegaard JI, Ricardo-Gonzalez RR, Red Eagle A, Vats D, Morel CR, et al (2008) Alternative M2 activation of Kupffer cells by PPARdelta ameliorates obesity-induced insulin resistance. Cell Metab 7: 496–507. 18522831 10.1016/j.cmet.2008.04.003 PMC2587370

[47] Kang K, Reilly SM, Karabacak V, Gangl MR, Fitzgerald K, et al (2008) Adipocyte-derived Th2 cytokines and myeloid PPARdelta regulate macrophage polarization and insulin sensitivity. Cell Metab 7: 485–495. 18522830 10.1016/j.cmet.2008.04.002 PMC2586840

[48] Brand K, Page S, Rogler G, Bartsch A, Brandl R, et al (1996) Activated transcription factor nuclear factor-kappa B is present in the atherosclerotic lesion. J Clin Invest 97: 1715–1722. 8601637 10.1172/JCI118598 PMC507236

[49] Wilson SH, Caplice NM, Simari RD, Holmes DR Jr, Carlson PJ, et al (2000) Activated nuclear factor-kappaB is present in the coronary vasculature in experimental hypercholesterolemia. Atherosclerosis 148: 23–30. 10580167 10.1016/s0021-9150(99)00211-7

[50] Van der Heiden K, Cuhlmann S, Luong le A, Zakkar M, Evans PC (2010) Role of nuclear factor kappaB in cardiovascular health and disease. Clin Sci (Lond) 118: 593–605. 20175746 10.1042/CS20090557

[51] Tikellis C, Jandeleit-Dahm KA, Sheehy K, Murphy A, Chin-Dusting J, et al (2008) Reduced plaque formation induced by rosiglitazone in an STZ-diabetes mouse model of atherosclerosis is associated with downregulation of adhesion molecules. Atherosclerosis 199: 55–64. 18093596 10.1016/j.atherosclerosis.2007.10.038

[52] Jialal I, Devaraj S (2001) Inflammation and atherosclerosis: the value of the high-sensitivity C-reactive protein assay as a risk marker. Am J Clin Pathol 116 Suppl: S108–115 11993695 10.1309/J63V-5LTH-WYFC-VDR5

